# Supplementary material for: Tracheal terminal cells of Drosophila are immune privileged to maintain their Foxo-dependent structural plasticity
Source: eLife. 2026 Jun 17;13:RP102369. doi: 10.7554/eLife.102369 (PMC13275064; doi:10.7554/eLife.102369)
Supplement: MDAR checklist [file elife-102369-mdarchecklist1.docx]

**Materials Design Analysis Reporting (MDAR)**

**Checklist for Authors**

The [MDAR framework](https://osf.io/xfpn4/) establishes a minimum set of requirements in transparent reporting mainly applicable to studies in the life sciences.

*eLife* asks authors to **provide detailed information within their article** to facilitate the interpretation and replication of their work. Authors can also upload supporting materials to comply with relevant reporting guidelines for health-related research (see [EQUATOR Network](http://www.equator-network.org/%20)), life science research (see the [BioSharing Information Resource](http://biosharing.org/)), or animal research (see the [ARRIVE Guidelines](http://www.plosbiology.org/article/info:doi/10.1371/journal.pbio.1000412) and the [STRANGE Framework](https://doi.org/10.1038/d41586-020-01751-5); for details, see *eLife*’s [Journal Policies](https://reviewer.elifesciences.org/author-guide/journal-policies)). Where applicable, authors should refer to any relevant reporting standards materials in this form.

For all that apply, please note **where in the article** the information is provided. Please note that we also collect information about data availability and ethics in the submission form.

**Materials:**

| **Newly created materials** | **Indicate where provided: section/figure legend** | **N/A** |
| --- | --- | --- |
| The manuscript includes a dedicated "materials availability statement" providing transparent disclosure about availability of newly created materials including details on how materials can be accessed and describing any restrictions on access. |  | x |
|  |  |  |
| **Antibodies** | **Indicate where provided: section/figure legend** | **N/A** |
| anti-Dcp-1 (Cell Signaling Technology; Cat#9578) | 181-188; Figure 4; Figure 4SuppFig. 2 |  |
| anti-pJNK (Promega; Cat#V7931) | 214-217; Figure 5 |  |
| anti-Relish (Developmental Studies Hybridoma Bank (DSHB); Cat#anti-Relish-C 21F3) | 212-214; Figure 5 |  |
| anti-GFP mouse (Developmental Studies Hybridoma Bank (DSHB); Cat#DSHB-GFP-8H11) | 182-184; Figure 4; Figure 5 |  |
| anti-GFP rabbit (Sigma-Aldrich; Cat# SAB4701015) | Figure 5 |  |
| Goat anti-Rabbit IgG, AF488 (Invitrogen; Cat#A-11034) | Figure 5 |  |
| Goat Anti-Mouse IgG, AF 488 (Jackson ImmunoResearch Laboratories; Cat#115-545-003) | 182-184; Figure 4; Figure 5 |  |
| Goat Anti-Rabbit IgG, Cy3 (Jackson ImmunoResearch Laboratories; Cat#111-165-003) | 181-188; 214-217; Figure 4; Figure 4SuppFig. 2; Fig. 5 |  |
| Goat Anti-Mouse IgG (Jackson ImmunoResearch Laboratories; Cat#115-165-003) | 212-214; Figure 5 |  |
|  |  |  |
| **DNA and RNA sequences** | **Indicate where provided: section/figure legend** | **N/A** |
| Short novel DNA or RNA including primers, probes: Sequences should be included or deposited in a public repository. |  | x |
|  |  |  |
| **Cell materials** | **Indicate where provided: section/figure legend** | **N/A** |
| Cell lines: Provide species information, strain. Provide accession number in repository OR supplier name, catalog number, clone number, OR RRID. |  | x |
| Primary cultures: Provide species, strain, sex of origin, genetic modification status. |  | x |
|  |  |  |
| **Experimental animals** | **Indicate where provided: section/figure legend** | **N/A** |
| *Drosophila melanogaster* strains were obtained from the Bloomington Drosophila Stock Center or from published sources and collaborators. (see provided Key Resource Table). Larvae of the late 3^rd^ instar stage were used for all experiments. It was not distinguished between male and female larvae. | Used throughout the whole study. |  |
|  |  |  |
| **Plants and microbes** | **Indicate where provided: section/figure legend** | **N/A** |
| *Pectobacterium carotovorum* (Ecc-15, 2141) was used for infection experiments. | Figure 1 and 2 |  |
|  |  |  |
| **Human research participants** | **Indicate where provided: section/figure legend) or state if these demographics were not collected** | **N/A** |
| If collected and within the bounds of privacy constraints report on age, sex, gender and ethnicity for all study participants. |  | **x** |

**Design:**

| **Study protocol** | **Indicate where provided: section/figure legend** | **N/A** |
| --- | --- | --- |
| If the study protocol has been pre-registered, provide DOI. For clinical trials, provide the trial registration number OR cite DOI. |  | x |
|  |  |  |
| **Laboratory protocol** | **Indicate where provided: section/figure legend** | **N/A** |
| Provide DOI OR other citation details if detailed step-by-step protocols are available. |  | x |
|  |  |  |
| **Experimental study design (statistics details) *** | | |
| **For in vivo studies: State whether and how the following have been done** | **Indicate where provided: section/figure legend. If it could have been done, but was not, write “not done”** | **N/A** |
| Sample size determination |  | x |
| Randomisation |  | x |
| Blinding |  | x |
| Inclusion/exclusion criteria |  | x |
|  |  |  |
| **Sample definition and in-laboratory replication** | **Indicate where provided: section/figure legend** | **N/A** |
| Infection experiments: Each animal describes one biological replicate and one data point. The infection was replicated at least three times using several independent vials with larvae. Two controls groups were used. (1) Larvae without any treatment, (2) larvae that were treated with the bacterial growth medium (LB) but without bacteria. Only one control group is shown in the figures. | Figure 1, 2, 3,  Figure 1-Figure Supplement 1 |  |
| TTC branch quantification: F1 third instar larvae from at least three independent crosses were used for image acquisition. One TTC per animal was defined as one biological replicate. Image acquisition took place on different days. All images of one group were pooled and used for branch quantification. | Figure 4, 5, 6, 7 |  |
| Hypoxia sensitivity measurement: F1 third instar larvae from at least three independent crosses were used. One biological replicate was defined as one vial with 10 (Figure 4-Figure supplement 3)/20 (Figure 4) animals. The experiment was replicated at least six times. | Figure 4, Figure 4-Figure supplement 3 |  |
| Epithelial thickness: Each data point represents one third instar larva. At least 10 larvae per group were analyzed.  The experiment was independently replicated three times using independent vials. Image acquisition was performed on different days. No samples were excluded from the analysis | Figure 4-Figure supplement 1 |  |
| Fluorescence quantification: Each trachea dissected from one animal describes one biological replicate and one data point. The immunohistochemical staining was performed using larvae from several independent vials. | Figure 4-Figure supplement 3 |  |
|  |  |  |
| **Ethics** | **Indicate where provided: section/submission form** | **N/A** |
| Studies involving human participants: State details of authority granting ethics approval (IRB or equivalent committee(s), provide reference number for approval. |  | x |
| Studies involving experimental animals: State details of authority granting ethics approval (IRB or equivalent committee(s), provide reference number for approval. | Ethics approval not required for *Drosophila melanogaster*. |  |
| Studies involving specimen and field samples: State if relevant permits obtained, provide details of authority approving study; if none were required, explain why. |  | x |
|  |  |  |
| **Dual Use Research of Concern (DURC)** | **Indicate where provided: section/submission form** | **N/A** |
| If study is subject to dual use research of concern regulations, state the authority granting approval and reference number for the regulatory approval. |  | x |

**Analysis:**

| **Attrition** | **Indicate where provided: section/figure legend** | **N/A** |
| --- | --- | --- |
| TTC branch quantification: For some TTC branch images, the image quality (GFP expression) was too bad for quantification and use with NeuronJ, these images were excluded from quantification. For the TTCs with a strong phenotype (e.g. PGRP-LCxOE, kayOE;JraOE) the analysis was accordingly challenging with a higher number of excluded images in these groups. | Figure 4, 5, 6 |  |
|  |  |  |
| **Statistics** | **Indicate where provided: section/figure legend** | **N/A** |
| All data from each group was tested for normal distribution (Shapiro-Wilk test). If the data were not normally distributed, the non-parametric Mann-Whitney U test was used, otherwise an unpaired t-test was used. Within one graph, the same test was applied. Statistical analysis was performed with GraphPad Prism. | Figure 4, 5, 6, 7  Figure S2, S3 |  |
| Data from TTC branch quantification were tested for outliers with GraphPad Prism Outlier test (ROUT (Q = 1 %). Outliers were excluded from the analysis. You can find all data including the outliers in the available data table. | Figure 4, 5, 6 |  |
|  |  |  |
| **Data availability** | **Indicate where provided: section/submission form** | **N/A** |
| For newly created and reused datasets, the manuscript includes a data availability statement that provides details for access (or notes restrictions on access). |  | x |
| When newly created datasets are publicly available, provide accession number in repository OR DOI and licensing details where available. | Deposited on Dryad (https://doi.org/10.5061/dryad.1rn8pk19h) |  |
| If reused data is publicly available provide accession number in repository OR DOI, OR URL, OR citation. |  | x |
|  |  |  |
| **Code availability** | **Indicate where provided: section/figure legend** | **N/A** |
| For any computer code/software/mathematical algorithms essential for replicating the main findings of the study, whether newly generated or re-used, the manuscript includes a data availability statement that provides details for access or notes restrictions. |  | x |
| Where newly generated code is publicly available, provide accession number in repository, OR DOI OR URL and licensing details where available. State any restrictions on code availability or accessibility. |  | x |
| If reused code is publicly available provide accession number in repository OR DOI OR URL, OR citation. |  | x |

**Reporting:**

The MDAR framework recommends adoption of discipline-specific guidelines, established and endorsed through community initiatives.

| **Adherence to community standards** | **Indicate where provided: section/figure legend** | **N/A** |
| --- | --- | --- |
| State if relevant guidelines (e.g., ICMJE, MIBBI, ARRIVE, STRANGE) have been followed, and whether a checklist (e.g., CONSORT, PRISMA, ARRIVE) is provided with the manuscript. |  | x |

* We provide the following guidance regarding transparent reporting and statistics; we also refer authors to [Ten common statistical mistakes to watch out for when writing or reviewing a manuscript](https://doi.org/10.7554/eLife.48175).

**Sample-size estimation**

- You should state whether an appropriate sample size was computed when the study was being designed
- You should state the statistical method of sample size computation and any required assumptions
- If no explicit power analysis was used, you should describe how you decided what sample (replicate) size (number) to use

**Replicates**

- You should report how often each experiment was performed
- You should include a definition of biological versus technical replication
- The data obtained should be provided and sufficient information should be provided to indicate the number of independent biological and/or technical replicates
- If you encountered any outliers, you should describe how these were handled
- Criteria for exclusion/inclusion of data should be clearly stated
- High-throughput sequence data should be uploaded before submission, with a private link for reviewers provided (these are available from both GEO and ArrayExpress)

**Statistical reporting**

- Statistical analysis methods should be described and justified
- Raw data should be presented in figures whenever informative to do so (typically when N per group is less than 10)
- For each experiment, you should identify the statistical tests used, exact values of N, definitions of center, methods of multiple test correction, and dispersion and precision measures (e.g., mean, median, SD, SEM, confidence intervals; and, for the major substantive results, a measure of effect size (e.g., Pearson's r, Cohen's d)
- Report exact p-values wherever possible alongside the summary statistics and 95% confidence intervals. These should be reported for all key questions and not only when the p-value is less than 0.05.

**Group allocation**

- Indicate how samples were allocated into experimental groups (in the case of clinical studies, please specify allocation to treatment method); if randomization was used, please also state if restricted randomization was applied
- Indicate if masking was used during group allocation, data collection and/or data analysis
