# Supplementary material for: Tracheal terminal cells of Drosophila are immune privileged to maintain their Foxo-dependent structural plasticity
Source: eLife. 2026 Jun 17;13:RP102369. doi: 10.7554/eLife.102369 (PMC13275064; doi:10.7554/eLife.102369)
Supplement: Peer review history

# Editor report: eLife Assessment

**DOI:** 10.7554/eLife.102369.3.sa0

**Authors:** Jiwon Shim

**Affiliations:** Seoul National University Republic of Korea

**Keywords:** Convincing, Solid, Valuable

This is a **valuable** report describing tracheal terminal cells (TTCs) in *Drosophila* as an immune-privileged organ. The authors demonstrated that TTCs lack expression of the membrane-associated peptidoglycan recognition receptor PGRP-LC, which protects these cells from immune pathway activation and JNK-mediated cell death to maintain TTC homeostasis. While the genetic experiments using RNAi and overexpression are **convincing** and **solid**, the broader biological significance of this phenomenon requires further investigation. This work will be of interest to researchers in innate immunity across various model systems.

# Referee report: Reviewer #1 (Public review)

**DOI:** 10.7554/eLife.102369.3.sa1

Summary:

In their manuscript entitled "Terminal tracheal cells of Drosophila are immune privileged to maintain their Foxo-dependent structural plasticity", Bossen and colleagues determine that the terminal cells of the tracheal system differ from other larval tracheal cells in that they do not typically show an Imd-dependent immune response to fungal and viral infections. Authors reach this conclusion based on the expression of a reporter line, Drs-GFP. The authors speculate that this difference may reflect differential expression of an immune pathway component, as tracheal terminal cells (ttcs) do not respond to forced expression of PRGP-LS. The authors then go on to show that, unlike the other cells of the tracheal system, terminal cells do not express PGRP-LC as reported by a GAL4 enhancer trap. Forced expression of PGRP-LC in terminal cells resulted in reduced branching, cell damage and features of the cell death program. These effects could be suppressed by depletion of AP-1 or Foxo transcription factors. Authors show that Foxo plays a negative role in branching of ttcs, with ectopic branching occurring upon RNAi (or under hypoxic conditions). The authors speculate that immune privilege of the ttcs may have evolved to permit Foxo regulation of ttc branching.

Strengths:

The authors provide compelling genetic data that support their overall conclusions.

Weaknesses:

FC do not appear to express DRS reporter in Figure 1 or elsewhere, raising the question of whether fusion cells are also immune privileged.

Fig 5, TRE_RFP expression, is convincing in wt ttc, but not in ttc o/x PGRP-LCx

# Referee report: Reviewer #2 (Public review)

**DOI:** 10.7554/eLife.102369.3.sa2

Summary:

In this study, Bossen et al. looked at the immune status of the tracheal terminal cells (TTCs) in Drosophila larvae. The authors propose that these cells do show PGFP-LCx expression and, hence, lack immune function. Artificial overexpression of the PGRP-LCx in the TTCs causes these cells to undergo apoptosis.

Strengths:

Only a few groups have tried to look at the immune status of the trachea, though we know that AMPs are expressed there after infection. This exciting study attempts to understand the differences in the tracheal cells that do not produce AMPs upon infection.

Weaknesses:

The reason why the TTCs have some immune privilege still needs to be completely clear. Whether the phenotype is cell autonomous or contributes to the cellular immune system is not evaluated. As we know, crystal cells also maintain oxygen levels in larvae; whether in the absence of a terminal trachea, the crystal cells have any role is not explored.

My particular comments on the figures are as follows:

(1) In Figure 2, the PGRP-LCx signal should be quantified as done for Drosomycin GFP, as shown in Figure 1.

- The authors have now done this.

(2) In Fig 2F and G are the larvae infected? If not, what happens to PGRP-LCx expression post Ecc15 infection?

- The authors have answered this question, saying infection has no effect on TTCs' Dr-GFP expression.

(3) Is the effect of overexpression of LCx exaggerated post-infection? In particular, when it comes to the escape phenotype.

- This was not done; the infection experiment was done with PGRP-LE overexpression.

(4) Does overexpression of anti-apoptotic genes in TTC and PGRP-LCx rescue the TTC branching?

- This was not done.

(5) Have the authors tried to rescue the larvae with shallow food?

- This was not done.

(6) Is there any effect on the circulating hemocytes or lymph gland in the PGFRP-LCx overexpressing animals?

- This was not done.

# Referee report: Reviewer #3 (Public review)

**DOI:** 10.7554/eLife.102369.3.sa3

Summary:

The authors report that tracheal terminal cells (TTCs) in Drosophila do not activate innate immunity following bacterial infection, and attribute this to the absence of PGRP-LCx expression in these cells. Forced activation of the Imd pathway in TTCs leads to JNK-mediated cell death and reduced tracheal branching. The authors propose that this immune-privileged status preserves Foxo-dependent structural plasticity, which is essential for TTCs to respond to changing environmental conditions such as hypoxia.

Strengths:

The revised manuscript represents a meaningful improvement over the initial submission. The addition of multiple antimicrobial peptide reporters substantially strengthens the key observation that TTCs do not mount a humoral immune response upon infection, moving beyond reliance on the Drs-GFP reporter alone. The mechanistic dissection of the cell death pathway - demonstrating roles for JNK, AP-1, and Foxo downstream of ectopic PGRP-LCx activation - is well-executed and provides solid mechanistic insight. The inclusion of a second, independent UAS-PGRP-LCx line with a milder phenotype adds useful calibration. The hypoxia sensitivity assays provide physiological context, and the discussion of the gradient hypothesis, while based on qualitative observation, is logically reasoned and addresses a legitimate alternative interpretation.

Weaknesses:

The primary remaining concern is that the absence of PGRP-LCx expression in TTCs is supported by a single GAL4 enhancer trap line, without independent validation by complementary methods such as in situ hybridization, antibody staining, or reanalysis of publicly available single-cell transcriptomic data. The authors acknowledge this limitation transparently. While the convergent evidence from infection experiments - in which neither the Drs-GFP reporter nor the PGRP-LCx-Gal4 line shows TTC activation - lends indirect support, orthogonal confirmation would more definitively establish this mechanistic claim.

Additionally, the finding that Dcp-1 cleavage occurs in non-TTC tracheal cells as well suggests that Imd-mediated apoptotic signaling is not uniquely restricted to TTCs, and the Discussion could more explicitly address what distinguishes the TTC response in terms of degree or cellular context.

# Author comment: Author response

**DOI:** 10.7554/eLife.102369.3.sa4

**Authors:** Judith M Bossen, Larissa Fritz, Reshmi Raveendran, Leizhi Shi, Jingjing He, Thomas Roeder

**Affiliations:** Kiel University Kiel Germany; Kiel University Kiel Germany; Kiel University Kiel Germany; Linyi People's Hospital Linyi, Shandong China; Kiel University Kiel Germany; Kiel University Kiel Germany

The following is the authors’ response to the original reviews.

**Public Reviews:**

**Reviewer #1 (Public review):**

Summary:

In their manuscript entitled "Terminal tracheal cells of Drosophila are immune privileged to maintain their Foxo-dependent structural plasticity", Bossen and colleagues determine that the terminal cells of the tracheal system differ from other larval tracheal cells in that they do not typically show an Imd-dependent immune response to fungal and viral infections. The authors reach this conclusion based on the expression of a reporter line, Drs-GFP. The authors speculate that this difference may reflect differential expression of an immune pathway component, as tracheal terminal cells (TTCs) do not respond to forced expression of PRGP-LS. The authors then go on to show that, unlike the other cells of the tracheal system, terminal cells do not express PGRP-LC as reported by a GAL4 enhancer trap. Forced expression of PGRP-LC in terminal cells resulted in reduced branching, cell damage, and features of the cell death program. These effects could be suppressed by the depletion of AP-1 or Foxo transcription factors. The authors show that Foxo plays a negative role in the branching of TTCs, with ectopic branching occurring upon RNAi (or under hypoxic conditions). The authors speculate that the immune privilege of the TTCs may have evolved to permit Foxo regulation of TTC branching.

Strengths:

The authors provide compelling genetic data.

Weaknesses:

(1) The authors state that after infection 34% of larvae were not GFP+ as defined by the detection of Drs-GFP in dorsal branches. The authors should clarify if these larvae are completely without response to infection, with no Drs-GFP in dorsal trunks and or other tracheal branches. If these larvae are entirely unresponsive, could authors indicate why this might be? Also, at this point in the manuscript, the authors are somewhat misleading regarding TTC expression of Drs-GFP - they should state at this point that there are some TTCs that do express Drs-GFP, and also should address their prior study of Drs-GFP induction which does not claim exclusion of TTC Drs-GFP expression.

GFP– indicates the absence of detectable fluorescence in regions proximal to the TTCs (dorsal branch and fusion cells). Our analysis specifically focused on these regions and did not assess fluorescence in other parts of the tracheal system. Therefore, the reported 34% of larvae classified as GFP– does not imply a complete absence of response in these animals; rather, no fluorescence was detected within our defined region of interest. To clarify how fluorescence in TTCs was quantified, we have added a schematic (new Fig. 1F). In addition, new Fig. S1 illustrates that AMP reporter activation frequently occurs in other tissues.

Our observations are consistent with earlier reports. In the original description of the AMP reporter lines, Tzou et al. (2000; https://doi.org/10.1016/S1074-7613(00)00072-8) reported that “only a fraction of the flies or larvae exhibited fluorescence in surface epithelia, and the proportion of GFP-expressing animals was variable from one culture vial to the next. In addition, fluorescence was rarely distributed throughout the whole tissue and was limited to restricted areas of the epithelium,” suggesting that AMP reporter activation can occur locally rather than uniformly across tissues.

In a previous study (https://doi.org/10.1186/1471-2164-9-446), we reported that airway epithelial cells, including the finest tracheal endings on target organs, can activate drosomycin transcription following infection. However, that study focused specifically on infected larvae. Importantly, it did not quantify the frequency of reporter activation or analyze TTC-specific phenotypes. As such, those statements should not be interpreted as implying uniform or ubiquitous reporter activation across all tracheal cells.

(2) The authors describe the terminal cell phenotype as "shrunken" but this implies loss of size or pruning, however, it is not clear whether the defects could equally be due to lack of growth or slower growth.

We omitted the term “shrunken” in the present manuscript to avoid potential misinterpretation.

(3) Figure 1 suggests that GFP+ dorsal branches are not uniform in their expression of Drs-GFP, it seems more patchy. The authors should define the fraction of dorsal branch cells that are Drs-GFP positive. Also, are fusion cells Drs-GFP positive?

We included a schematic illustrating our quantification approach (new Fig. 1F). We also revised the wording to clarify that GFP^+^ animals include fluorescence not only in the dorsal branch (DB) but also in fusion cells (FCs), i.e., structures located between the dorsal trunks and the terminal tracheal cells (TTCs). Any structure in proximity to the TTCs that shows GFP expression was scored as GFP^+^. In most cases, GFP expression was observed in the dorsal fusion cells.

(4) Drs-GFP expression is largely absent from terminal cells; however, a still significant # of terminal cells show expression (8%). Authors argue that PRGP-LC expression is absent based on a GAL4 transgenic line. If this line reflects endogenous PRGP-LC expression, should there not be 8% positive TTCs? Or is the 8% Drs-GFP expression independent of the IMD receptor?

We detected PGRP-LE expression in approximately 3% of epithelial tracheal cells that expressed Drs after infection (Fig. 3F,G). This observation suggests that Drs activation can occur through a mechanism independent of PGRP-LCx. We have incorporated this finding into both the Results and Discussion sections.

(5) Figure 2: the authors state that TTCs are negative even with induced PRGP-LE expression - should there not be at least 8% that are positive?

We included infection of the PGRP-LE overexpression and could see Drs-GFP expression in 3 % of the cases, which we did not see without infection.

(6) The authors compare PRGP-LC expression to induction of cell death by expression of reaper and hid. Reaper and Hid had stronger effects and eliminated TTCs. See cleavage of caspase Dpc-1 in PRGP-LC expressing cells. Is caspase cleavage always diagnostic of apoptosis or could the weaker than rpr/hid phenotype imply a different function?

We have included the potential non-apoptotic functions of Dcp-1 in the Discussion. The weaker phenotype observed could therefore be explained by a non-apoptotic role of Dcp-1.

(7) Drs-GFP expression is said to be "completely" absent from tracheal terminal cells when the entire tracheal system is expressing PGRP-LE.

We have revised the wording accordingly.

(8) Figure 5, TRE_RFP expression, is not convincing that it is higher or in terminal cells. https://doi.org/10.7554/eLife.102369.1.sa2

We have revised the wording in line 230.

**Reviewer #2 (Public review):**

Summary:

In this study, Bossen et al. looked at the immune status of the tracheal terminal cells (TTCs) in Drosophila larvae. The authors propose that these cells do show PGFP-LCx expression and, hence, lack immune function. Artificial overexpression of the PGRP-LCx in the TTCs causes these cells to undergo apoptosis.

Strengths:

Only a few groups have tried to look at the immune status of the trachea, though we know that AMPs are expressed there after infection. This exciting study attempts to understand the differences in the tracheal cells that do not produce AMPs upon infection.

Weaknesses:

The reason why the TTCs have some immune privilege still needs to be completely clear. Whether the phenotype is cell autonomous or contributes to the cellular immune system is not evaluated. As we know, crystal cells also maintain oxygen levels in larvae; whether in the absence of terminal trachea, the crystal cells have any role is not explored. https://doi.org/10.7554/eLife.102369.1.sa1

In addition to the Drs-GFP reporter line, we performed new infection experiments using additional antimicrobial peptide reporters to further support our observations. While these experiments confirm the humoral immune response, they do not address the mechanisms underlying the apparent immune privilege. Our analysis therefore focuses specifically on the humoral immune response and does not allow conclusions regarding potential contributions of the cellular immune system, including crystal cells, to maintaining oxygen levels in animals with impaired TTCs. Notably, complete loss of TTCs is lethal, as demonstrated by TTC ablation using hid;rpr expression (Fig. 4F).

**Reviewer #3 (Public review):**

Summary:

The authors report that tracheal terminal cells (TTCs) in Drosophila do not activate innate immunity following bacterial infection. They attribute this to the lack of expression of PGRP-LCx in these cells. Forced activation of the Imd pathway in TTCs leads to cell death and a reduction in tracheal branching. The authors propose a mechanism for cell death induction via pathways involving JNK, AP-1, and foxo. They suggest that the suppression of innate immunity in TTCs may serve to maintain their plasticity, preparing them for responses to hypoxic conditions.

Strengths:

(1) The study addresses the understudied area of immune privilege in innate immunity, providing a potentially important example in Drosophila TTCs.

(2) The molecular characterization of the cell death pathway induced by forced Imd activation is well-executed and provides solid mechanistic insights.

(3) The authors draw interesting parallels between Drosophila TTCs and mammalian endothelial cells, suggesting broader implications for their findings.

Weaknesses:

(1) The core premise of the study - that TTCs do not activate innate immunity following bacterial infection - relies heavily on a single readout (Drs reporter). Additional markers of immune activation would strengthen this crucial claim.

We included new experiments using additional antimicrobial peptide reporter genes that show results similar to those obtained with the Drs-GFP reporter (new Fig. 1).

(2) The evidence for the lack of PGRP-LCx expression in TTCs is based on a single GAL4 reporter line. Given the importance of this observation to the authors' model, validation using alternative methods would be beneficial.

Although we were not able to include alternative methods to further confirm our hypothesis, we performed additional infection experiments. Upon bacterial infection, we observed a strong increase in GFP fluorescence throughout the animal and in many other tissues, while still detecting no response in the TTCs. These results further support our hypothesis.

(3) The phenotypes observed upon forced activation of the Imd pathway in TTCs, while intriguing, may be influenced by non-physiological levels of pathway activation. The authors should address this potential caveat and consider examining the effects of more moderate pathway activation. https://doi.org/10.7554/eLife.102369.1.sa0

We used two independent UAS-PGRP-LCx lines located on different chromosomes. One line (III) produced a stronger phenotype than the other (II). We clarified this point in the Results section (Fig. 4C,D) and added supplementary data (new Fig. S2) showing that both lines produce comparable phenotypes when expressed using an alternative tracheal driver. The epithelial thickening observed follows the same pattern as the phenotype detected in TTCs, indicating that even moderate pathway activation leads to similar effects. However, we acknowledge that this represents ectopic pathway activation and therefore likely reflects a non-physiological level of signaling.

**Recommendations for the authors:**

**Reviewer #2 (Recommendations for the authors):**

My particular comments on the figures are as follows:

(1) In Figure 2, the PGRP-LCx signal should be quantified as done for Drosomycin GFP, as shown in Figure 1.

We agree and have added a quantification.

(2) In Figure 2F and G are the larvae infected? If not, what happens to PGRP-LCx expression post Ecc15 infection?

We also included infected larvae to test whether infection induces GFP expression in TTCs. However, GFP expression was never observed in TTCs, although overall fluorescence increased in other tissues.

(3) Is the effect of overexpression of LCx exaggerated post-infection? In particular when it comes to the escape phenotype.

We induced mild Imd pathway activation by expressing PGRP-LE using a tracheal driver active in all tracheal cells, including TTCs, for 24 hours. In addition, these larvae were infected and their sensitivity to hypoxia was assessed. Animals expressing PGRP-LE in the trachea showed increased sensitivity to hypoxia, which was further enhanced following infection.

(4) Does overexpression of anti-apoptotic genes in TTC and PGRP-LCx rescue the TTC branching?

This point was not addressed.

(5) Have the authors tried to rescue the larvae with shallow food?

This point was not addressed.

(6) Is there any effect on the circulating hemocytes or lymph glands in the PGFRP-LCx overexpressing animals?

This point was not addressed.

**Reviewer #3 (Recommendations for the authors):**

The authors present an intriguing model of immune privilege in Drosophila tracheal terminal cells (TTCs). This model is built upon three key pillars: (1) the absence of innate immune activation in TTCs, (2) the lack of PGRP-LCx expression in TTCs, and (3) the induction of cell death when innate immunity is activated in TTCs. However, the experimental evidence supporting each of these critical points requires substantial strengthening. The reviewer recommends the following improvements and additional experiments to address these core issues:

(1) Innate immune activation in TTCs:

Evaluate the expression of additional antimicrobial peptide reporters to provide a more comprehensive assessment of innate immune activation in TTCs.

In addition to the Drs-GFP reporter line, we performed new infection experiments using other antimicrobial peptide reporters to confirm our results.

(2) PGRP-LCx expression in TTCs:

Validate the PGRP-LCx-GAL4 line used in the study to ensure it accurately reflects endogenous PGRP-LCx expression.

Employ complementary techniques such as in situ hybridization and antibody staining to corroborate the absence of PGRP-LCx in TTCs.

We also included infection experiments using PGRP-LCx-Gal4 larvae. Infection did not trigger GFP expression in TTCs. However, the overall PGRP-LCx expression pattern observed in other larval tissues supports that the results reflect endogenous PGRP-LCx expression.

(3) Cell death induction upon immune activation in TTCs:

Address the possibility that the observed cell death is an artifact of strong, forced Imd pathway activation. To do that,

perform control experiments activating the Imd pathway in non-TTC tracheal cells to determine if cell death is specific to TTCs.

Use broader tracheal drivers (e.g., ppk4-GAL4 or btl-GAL4) to activate the Imd pathway and verify if cell death is indeed restricted to TTCs.

We included results from PGRP-LCx overexpression using the tracheal driver ppk4-Gal4 and stained for the apoptosis marker Dcp-1 (new Fig. S3). We observed increased Dcp-1 signal in dorsal trunk cells, indicating that PGRP-LCx-mediated Dcp-1 cleavage is not restricted to TTCs.

Ideally, generate a transgenic line expressing physiological levels of PGRP-LCx in TTCs and demonstrate that bacterial infection induces cell death specifically in TTCs through the proposed pathway. The reviewer acknowledges the complexity of this experiment but believe it would significantly strengthen the authors' conclusions.

We did not generate a new transgenic line but instead used an alternative UAS-PGRP-LCx line (II), which exhibits a milder phenotype. This has now been clarified more prominently in the Results section (Fig. 4C,D). Additionally, we performed further experiments showing an epithelial thickening phenotype whose severity depends on the UAS-PGRP-LCx line used (new Fig. S2).

In addition to the above major points

(4) Quantitative data presentation:

Provide quantitative analyses for the results presented in Figures 2 and 3J-K to allow for a more rigorous evaluation of the data.

We included a quantitative analysis of the results shown in Fig. 2 (now presented in new Fig. 3). In addition, we added quantification of fluorescence in the TTCs of infected larvae.

(5) Alternative hypothesis:

Consider and address an alternative explanation for the lack of innate immune activation in TTCs: the potential gradient of bacterial ligands from proximal trachea to distal TTCs. If this hypothesis is correct, one might expect to see a gradient of Drs expression correlating with the distance from the proximal trachea. Addressing this possibility would strengthen the authors' proposed model.

We now included the following paragraph as part of the discussion section.

“An alternative explanation for the observed lack of an immune response in TTCs could be their maximal distance from the spiracles. In this scenario, a gradient of bacterial inducers along the tracheal system might be expected, resulting in a gradual decrease in immune activation from the spiracles toward the TTCs. However, this is not what we observed. In tracheae that displayed an immune response, the response was largely homogeneous along the entire length of the tracheal system, from the spiracles to the TTCs. Only at the transition to the TTCs did the immune response drop abruptly. This observation argues against the gradient hypothesis and suggests that TTCs are specifically excluded from the immune response.”
